# Measuring social norms of intimate partner violence to exert control over wife agency, sexuality, and reproductive autonomy: an item response modelling of the IPV-ASRA scale

**DOI:** 10.1186/s12978-023-01632-w

**Published:** 2023-06-14

**Authors:** Sabrina C. Boyce, Alexandra M. Minnis, Julianna Deardorff, Sandra I. McCoy, Sneha Challa, Nicole Johns, Sani Aliou, Mohamad Brooks, Abdoul-Moumouni Nouhou, Perman Gochyyev, Mark Wilson, Holly Baker, Jay G. Silverman

**Affiliations:** 1 Center on Gender Equity and Health, School of Medicine, University of California San Diego, 9500 Gilman Drive, La Jolla, CA 92093 USA; 2 Women’s Global Health Imperative, RTI International, 2150 Shattuck Ave. Ste 800, Berkeley, CA 94704 USA; 3 Community Health Sciences, School of Public Health, University of California Berkeley, 2121 Berkeley Way, Berkeley, CA 94720-7360 USA; 4 Division of Epidemiology, School of Public Health, University of California Berkeley, 2121 Berkeley Way, Berkeley, CA 94720-7360 USA; 5 School of Nursing, University of San Francisco, 3333 California Street, San Francisco, CA 94118 USA; 6 Niger Country Office, Pathfinder International, Niamey, Niger; 7 Pathfinder International, 9 Galen Street, Suite 217, Watertown, MA 02472 USA; 8 GRADE Africa, Rue KK-138, Kouara Kano, BP 189, Niamey, Niger; 9 Graduate School of Education, University of California, 2121 Berkeley Way, Berkeley, CA 94720-1670 USA

**Keywords:** Social norms, Psychometrics, Intimate partner violence, Female agency, Reproductive autonomy, Measurement, Item response theory

## Abstract

**Background:**

The field of violence prevention research is unequivocal that interventions must target contextual factors, like social norms, to reduce gender-based violence. Limited research, however, on the social norms contributing to intimate partner violence or reproductive coercion exists. One of the driving factors is lack of measurement tools to accurately assess social norms.

**Methods:**

Using an item response modelling approach, this study psychometrically assesses the reliability and validity of a social norms measure of the acceptability of intimate partner violence to exert control over wife agency, sexuality, and reproductive autonomy with data from a population-based sample of married adolescent girls (ages 13–18) and their husbands in rural Niger (*n* = 559 husband-wife dyads) collected in 2019.

**Results:**

A two-dimensional Partial Credit Model for polytomous items was fit, showing evidence of reliability and validity. Higher scores on the “challenging husband authority” dimension were statistically associated with husband perpetration of intimate partner violence.

**Conclusions:**

This brief scale is a short (5 items), practical measure with strong reliability and validity evidence. This scale can help identify populations with high-need for social norms-focused IPV prevention and to help measure the impact of such efforts.

**Supplementary Information:**

The online version contains supplementary material available at 10.1186/s12978-023-01632-w.

## Plain language summary

Long-term prevention of gender-based violence, like intimate partner violence and reproductive coercion, requires efforts to change the social environment that facilitates violence against women, yet limited research is available on how to change social environments. One reason is that there are few tools to accurately measure social environments, including social norms, which are the unspoken rules about what behavior is acceptable and what behavior is not. The present research assessed a new social norms measurement tool on the acceptability of intimate partner violence to exert control over wife agency, sexuality, and reproductive autonomy using data from a population-based sample of married adolescents and their husbands in rural Niger (*n* = 559 husband-wife dyads) collected in 2019. We found that this scale had strong reliability and validity, and that the group of questions about challenging husband authority were related to husband perpetration of intimate partner violence against his wife. This brief scale is a short (5 questions), practical measure with strong reliability and validity evidence that can help identify populations with high-need for social norms-focused prevention and to help measure the impact of such efforts. This evidence strengthens the current set of measurement tools on social norms available to researchers and practitioners.

**Supplementary Information:**

The online version contains supplementary material available at 10.1186/s12978-023-01632-w.

## Background

Gender based violence, inclusive of intimate partner violence (IPV) and reproductive coercion (RC), is a pervasive global problem. IPV, which can include physical, sexual, and emotional violence perpetrated by a romantic partner, is experienced by one in three women worldwide [57]. Prevalence of RC, when a male partner interferes with a woman or girl’s efforts to control her fertility via pregnancy coercion or birth control sabotage [29, 30], ranges from 8.4% in the US [6], 10.2% in rural Niger [47], and 18.5% in Cote d’Ivoire [28]. IPV and RC often co-occur, with women who experience IPV at a much greater risk of RC than those who have not experienced IPV [48, 61]. Both IPV and RC have been found to be associated with a variety of negative consequences for health and wellbeing, including child bearing at younger ages, high parity, unintended pregnancy, depression, and HIV [18, 38, 46, 57], and are driven in large part by male partner beliefs of dominance and entitlement over female partners [11, 14]—beliefs that are shaped and reinforced by social norms [31].

Reducing IPV and RC is a primary focus of the United Nations 2030 Sustainable Development Goal 5 on gender equity. To achieve that goal, shifting social norms accepting of IPV and RC is critical [53]. Social norms, understood through social norms theory and the theory of interdependent action [26, 49], are the collective, often unspoken rules about what is normal and appropriate behaviour within a group of people; they are reinforced through social rewards and sanctions. For example, in highly patriarchal contexts, gender norms, a cultural template for how men and women should each behave, often drive behavioral expectations of dominant masculinity and female passivity, creating a social context that reinforces men’s violence against women [8, 12]. There are distinct constructs inherent in social norms theory. Descriptive norms are a person’s perception of what people in their community do in a given situation. Injunctive norms are a person’s perception of whether or not people in their community approve or disapprove of a certain behavior, and are reinforced by concerns around possible social sanctions. Second-order beliefs are a person’s perception of others’ opinions or beliefs about certain behaviors [26]. While many studies aggregate individual-level beliefs as a proxy for social norms, aggregated individual beliefs fail to capture key components of social norms: social expectations of others in the community, reference groups, and social sanctions and approval [26]. Few studies have examined injunctive norms or second order beliefs specific to IPV, but those that do suggest that perceived social norms and peer behaviour are related to individual IPV behaviour and that IPV can potentially be prevented by changing the social context [35, 43, 44, 51]. Violence prevention researchers are clear that effective interventions need to target social and contextual factors, like social norms, yet these efforts are substantially limited by lack of effective social norms measurement tools [5, 16, 24].

Few validated measures of IPV- or RC-related social norms exist. While some scales assess social norms broadly related to IPV and RC (e.g., traditional gender norms), very few scales measure the perceived social acceptability of these behaviors specifically or demonstrate correlation with violence perpetration. One IPV social norms measure, the Partner Violence Norms Scale, assesses gender norms and appropriate responses of family members to a woman experiencing IPV, but this only contains one item that assesses social acceptability of IPV behaviour itself (second-order belief) [13]. Another IPV social norms scale, the Social Norms and Beliefs about Gender Based Violence Scale, was validated for humanitarian settings in South Sudan and Somalia and contains a 4-item subscale (of 30-items) on motivations of wife beating (e.g., showing love, a husband’s right, and discipline), but has not been shown to be related to IPV behaviour [39]. Both scales offer a helpful measurement tool for understanding social norms that are broadly related to IPV, yet more validated measures assessing social norms directly related to the acceptability of IPV are needed.

Most studies attempting to measure IPV social norms have used proxy measures that aggregate individual-level attitudes about IPV, primarily using the Attitudes about IPV (ATT-IPV) scale [23, 25, 55, 59]. The ATT-IPV assesses individual-level attitudes regarding the justifiability of wife beating for behaviours representing a spectrum of gender transgressions (e.g., arguing with her husband) [60], a measure that has been integrated into the core Demographic and Health Survey with some variation across 90 countries [15]. Recognizing the limitations of aggregated individual-level attitudes, one study adapted the ATT-IPV scale to measure social norms by asking participants about the number of people in their village that would agree with each of the statements in the original ATT-IPV scale [50]. This adaptation of the ATT-IPV items to social norms items was not assessed for reliability or validity as a scale but could be a useful measure of second order beliefs if shown to be valid.

The current study seeks to assess the reliability and validity of a new measure of the social acceptability of IPV when it is used to exert control over wife agency, sexuality, and reproductive autonomy (IPV-ASRA Social Norms scale). Using data from a population-based sample of husbands of adolescent wives in rural Niger, we assessed these social norms by adapting the introductory question stem of the items in the ATT-IPV scale to transform them into a scale of second order beliefs about situations in which IPV are perceived to be socially acceptable. This study provides evidence of reliability and validity of the scale and tests the ability of the scale to differentiate between husbands who have and have not perpetrated IPV and RC. This measure maximizes efficiency and is primed for use in other contexts, given that it builds on the commonly used ATT-IPV scale in the DHS questionnaire that is used in many international contexts, contains a concise number of items, and helps address the need for social norms measurement tools.

## Methods

### Setting

The present psychometric analysis utilizes data from a cluster randomized control trial, called the *Reaching Married Adolescents Study in Niger* (RMA study)*,* that took place in the Dosso region of Niger. The Dosso region is made up of rural villages that identify as either culturally and linguistically Zarma or Hausa, each led by a male chief, where Islam and polygamy are widely practiced. While both Hausa and Zarma people are typically Islamic, patrilineal, and engage in agriculture-related subsistence, Hausa represent the majority cultural group in Niger and have younger ages at marriage for females relative to Zarma people [45]. In Niger, poverty, early and frequent childbearing, and gender inequity are pervasive. The overall birth rate is the highest in the world, with 6.8 births per woman, maternal mortality is high at 509 deaths per 100,000 live births, and 76% of women marry by the age of 18 [52, 54, 56]. Lifetime prevalence of physical IPV, sexual IPV, and RC are reported by 8.2%, 5.3%, and 10.2% of adolescent wives in the RMA study, respectively [47]. These estimates of IPV are lower than national estimates of IPV among adult women in other similar contexts who have experienced child marriage, likely related to the young age and short length of marriage at the time of surveying in this sample of adolescents [3].

### Sample and data collection

Cross-sectional data were collected as part of the third wave of data collection of the four-arm RMA cluster randomized control trial, which evaluated multiple community-based family planning interventions conducted from 2016 to 2019 to promote healthy birth spacing among married adolescents. Participants included husband and adolescent wife dyads; the present study primarily utilizes data from husbands to understand their perceived social norms around IPV, and how it relates to their behavior. A two-staged random sampling procedure was used at baseline in which 16 villages were randomly selected from the Dosso, Loga, and Doutchi districts of the Dosso region and within these selected villages, 25 households were randomly selected from a list of married adolescent girls provided by each village chief (*n* = 1042 husband-wife dyads at baseline). Inclusion criteria for villages included being Hausa or Zarma-speaking and having at least 1000 residents. Married adolescent girls were eligible for inclusion if they were (1) between the ages of 13–19 years at baseline (ethically old enough to consent to research and inclusive of married adolescents whose age estimation may be imprecise [37], (2) fluent in Hausa or Zarma, (3) planning to live in the village for the next 18 months and not be away > 6 months, and (5) willing and able to provide informed consent. Men were eligible if they were married to one of the eligible and successfully recruited girls. Selected households were visited up to three times; a randomly selected household replacement was used if unavailable. Due to low literacy, verbal informed consent administered by local data collection staff was obtained from all participants prior to participation in accordance with the Niger Ministry of Health recommendation. Guidelines from the World Health Organization on the ethical conduct of research on violence against women were used to develop study protocols [17]. More details on the study protocols have been published elsewhere [10].

Research assistants from Niger who were trained, fluent in French, Hausa, and Zarma, and gender-matched collected self-report quantitative data via interviews using pre-programmed tablet devices. Interviews were conducted in private locations of the participants’ choosing out of earshot from their spouse or other residents and required 40–60 min to complete. The third wave of data collection occurred in 2019, three years after baseline, and is the most recent data from this sample. The retention rate for male participants in the third wave was 52% relative to baseline, and attrition was associated with the husband traveling away from the village for more than 3 months in the past year (i.e., migrant worker), being younger in age, being younger at marriage, having a nulliparous wife, having a wife who was older at marriage, and having less education at baseline. Husband availability for data collection was likely impacted by annual patterns of seasonal migration for work among men after the season for agricultural work ends [22]. The retention rate for female participants in the third wave was 77% relative to baseline, and attrition was associated with having greater familial asset ownership at baseline. Participants received no compensation for their participation as any monetary amount was perceived as potentially coercive.

Our study was approved by both the University of California San Diego School of Medicine Institutional Review Board and the Research Ethics Board of the Niger Ministry of Health.

### Measure development

Based on a review of the literature and the expertise of the research team in IPV and RC, social norms, and social norms measurement, a 6-item scale was developed by adapting a common version of the ATT-IPV 5-item scale [60] (Table 1). Items within the ATT-IPV scale were adapted by adding a stem for second order beliefs that read, “People in your community believe that …” An additional item for the scale was developed and included that assessed the social acceptability of a husband hitting or beating his wife if she uses a family planning method without his permission (item 1 in the new IPV-ASRA Social Norms scale in Table 1), a form of controlling a wife’s reproductive autonomy. All items in this new IPV-ASRA Social Norms scale were polytomous with response options of “disagree”, “somewhat agree”, and “agree”, a reduced Likert scale due to the extremely low literacy of the sample. Based on feedback from pilot-testing and previous evidence from low-literacy populations indicating preference for a three option scale [9], this three-option response scale was used.

**Table 1:** Social norms scale regarding intimate partner violence to exert control over wife agency, sexuality, and reproductive autonomy (Reaching Married Adolescents Study, 2016–2019)

	Original ATT-IPV Scale	New Adapted IPV-ASRA Social Norms Scale
		*Preamble for all items: People in your community believe that…*
Item 1	N/A	…a husband is justified in hitting or beating his wife if she uses a family planning method without informing him
Item 2	A husband is justified in hitting or beating his wife if she refuses to have sex with him	…a husband is justified in hitting or beating his wife if she refuses to have sex with him
Item 3	If a woman refuses to have sex with her husband when he wants her to, he has the right to use force and have sex with her	…if a woman refuses to have sex with her husband when he wants her to, he has the right to use force and have sex with her
Item 4	A husband is justified in hitting or beating his wife if she argues with him	…a husband is justified in hitting or beating his wife if she argues with him
Item 5	A husband is justified in hitting or beating his wife if she goes out without telling him	…a husband is justified in hitting or beating his wife if she goes out without telling him
Item 6	A husband is justified in hitting or beating his wife if she burns his food	…a husband is justified in hitting or beating his wife if she burns his food

Response options for all IPV-ASRA Scale items: 0 = "Disagree", 1 = "Somewhat agree", 2 = "Agree"

All six items were reviewed by the in-country family planning intervention providers and the team of data collectors for contextual applicability and acceptability. The items were pilot tested with residents of the villages in which data collection took place (*n* = 15) by reading the items and response options to the pilot test participant individually in a private setting, confirming that the items were understood as intended, and the wording was clear. Aside from the need to reduce the number of response options noted above, no difficulties in understanding the items or negative impacts were reported.

The current analysis includes wives’ reports of IPV victimization, measured using items adapted from the DHS domestic violence module based on the WHO multi-country study [19], and RC victimization. IPV victimization was dichotomously assessed as an affirmative response to any of eight questions about whether their current husband has ever (1) “pushed you, shook you, or thrown something at you,” (2) “slapped you,” (3) “twisted your arm or pulled your hair,” (4) “hit you with his fist or with something that could hurt you,” (5) “kicked you, dragged you, or beat you up,” (6) “tried to choke you or burn you,” (7) “physically forced you to have sexual intercourse with him when you did not want to,” and (8) “physically forced you to perform any other sexual acts you did not want to.” Participants responding, “Don’t know”, “Decline to answer”, or had missing data for all items were considered missing. RC victimization was measured among wives using a RC victimization measure that was created for the cultural context of Niger using the research team’s expertise in RC, the expertise of in-country family planning providers and program facilitators, and the RC measure originally created by Miller et al. [29, 30, 47] (see Additional file 1 for details). RC victimization was also dichotomously assessed as an affirmative response to any of nine items regarding husband perpetration of pregnancy coercion or birth control sabotage.

### Analysis

The most recent wave of data (Wave 3) from husbands and wives was used in this analysis, as well as demographics from Wave 1. Our assessment of the IPV-ASRA Social Norms measure is grounded in item response theory, or item response modelling (IRM), which is an approach used in measurement science to assess scales intending to capture unobserved or latent constructs that cannot be directly measured, such as attitudes and beliefs [21]. We fit a Rasch model for polytomous items, called a Partial Credit Model (PCM) [27], to assess the reliability and validity of the IPV-ASRA Social Norms scale using ConQuest 4 software [2]. The PCM uses marginal maximum likelihood estimation to estimate a location parameter for each item, based on how “difficult” or “severe” it was for participants to respond “yes” to that item, relative to other items, allowing for the distance between each item to vary. The relative values of item location parameters are mapped relative to individuals’ scores centred at zero.

To assess the reliability of the measure, we calculated the expected a-posteriori (EAP) reliability index, a measure of reliability that compares the variance of the individual expected estimates of their perceived social acceptability of IPV, the latent construct, with the estimated total variance of the latent construct (EAP reliability generally considered acceptable if > 0.70) [32]. Internal structure validity was assessed in multiple ways. At the instrument level, we assessed reliability by using Wright Maps to plot the participants’ overall scores against each item’s difficulty (or “severity”) threshold to see if overall scores were approximately normally distributed and distribution of the scores of participants spanned the spread of the item difficulties. Using a PCM model, we obtained parameter estimates for each item’s difficulty threshold and weighted mean squared error fits (acceptable range of 0.67 and 1.33) [58]. We assessed for item bias (i.e., assessed to see if any particular item was more difficult or severe to respond “yes” to for some sub-groups of participants relative to others) by including item by group interaction terms to assess for differential item functioning (DIF) across Zarma and Hausa-speaking groups and treatment arms (evidence of item bias is generally considered > 0.426 logit difference in item functioning between subgroups) [36].

**Fig. 1:**
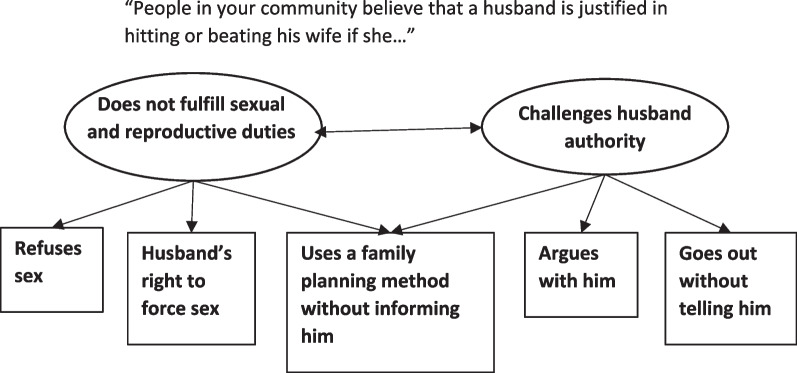
Hypothesized multidimensional taxonomy of the IPV-ASRA social norms scale

Based on content expertise, we hypothesized that the scale contained two subdimensions of the construct (Fig. 1). One dimension (items 1, 2, 3) represented the social acceptability of husband IPV if the wife does not fulfil the expected familial role around sexual and reproductive obligations (“Wifely sexual and reproductive duties”). Another dimension (items 1, 4, 5) represented the social acceptability of husband IPV if the wife challenged the authority of her husband (“Challenges husband authority”). To account for the two subdimensions of the construct, we fit a multidimensional PCM, a special case of a more general multidimensional random coefficients multinomial logit (MRCML) model [1] which allows for two latent sub-constructs to be considered. To see if each dimension performed better as separate scales, we also fit consecutive unidimensional PCMs separately for each dimension and compared the EAP reliabilities for each dimension with those given by the two-dimensional PCM [7].

Lastly, to obtain validity evidence based on relations to other related constructs, we hypothesized that male participants whose wives reported victimization of IPV and/or RC would be likely to perceive social norms more accepting of IPV in the situations described in the scale, compared to male participants whose wives did not. To test this, we fit two latent regression models [62]—one for wife reports of IPV victimization and one for RC victimization by her current husband—which allowed the means of the latent variable to vary across IPV or RC groups, respectively. To assess the scale’s relationship with IPV further, we ran a multiple-group multidimensional model to allow for each of three groups (those with affirmative reports of IPV, those without reports of IPV, and those with missing data on IPV) to have their own variance and correlations between dimensions. To ensure accurate comparisons across groups, we used the delta dimensional alignment technique so that all parameter estimates—across groups and dimensions—would be on a common metric [42]. MPlus statistical software was used for these analyses [34].

## Results

The sample contained 559 husband-wife dyads. The majority of participant households were culturally and linguistically Zarma (66%) (Table 2). At the third wave of data collection, husbands were on average 29.9 (range: 18–66) years old and wives were 20.4 (range: 16–22) years old. Most husbands had at least one child (range: 0–16 children) and one wife, however, 17% had more than one wife. Among wives, 9% reported ever having experienced IPV victimization by their current husband and 7% reported RC.

**Table 2:** Demographics at Wave 3 (Reaching Married Adolescents Study, *n* = 559)

	Mean (range)	*n* (%)
Cultural/linguistic group^a^		
Hausa		368 (66%)
Zarma		186 (34%)
Intervention participants^b^		412 (74%)
Husband age	29.9 (18–66)	
Husband age at marriage^a^	22.5 (12–53)	
Wife age	20.4 (16–22)	
Wife age at marriage^a^	14.2 (10–19)	
Length of marriage (years)^a^	3.1 (0–9)	
Husband education^a^		
Any government school		263 (47%)
Any Koranic school		203 (36%)
Both		79 (14%)
Neither		160 (29%)
Any paid work in past 12 months		
Husband		311 (56%)
Wife		104 (19%)
Wife number of children^a^	1.0 (0–5)	355 (64%)^c^
Husband number of children^a^	1.6 (0–16)	393 (70%)^c^
Husbands with > 1 wife		96 (17%)
Wife reported IPV victimization		50 (9%)
Wife reported RC victimization		39 (7%)

^a^Data collected at baseline

^b^Data were collected as part of a 4-arm cluster-randomized trial

^c^Number and percent who have at least one child

### Unidimensional partial credit model

Many husbands reported believing that people in their communities were not accepting of IPV in any of the circumstances included in the scale (40%). Crude scores on the scale ranged from the minimum to maximum scores (0 to 12) and had a mean of 2.8 (SD: 3.20), with higher scores indicating greater endorsement of acceptance of IPV. When the unidimensional PCM was fit, husbands’ scores across the IPV-ASRA social norms construct fell roughly in a normal distribution, indicating that the normality assumptions of the model were reasonable. Items and response category thresholds spanned across more extreme (i.e., higher) levels of the perceived social acceptability of IPV construct and did not adequately capture lower (i.e., less extreme) levels of the construct. In other words, the items in the scale mostly consisted of examples of peer perceptions of IPV that were severe or extreme and therefore difficult to endorse and contained few items that were less severe and therefore less difficult to endorse. The easiest item to endorse was the reproductive autonomy item (item 1; family planning use without informing the husband). The two hardest items to endorse were Item 6 (IPV for burning the food) and Item 3 (forcing sex if the wife refuses sex).

The DIF assessment across cultural/linguistic groups revealed that one of the most difficult items to endorse, IPV for burning the food, showed statistical bias (difference in item difficulty: 1.104) such that it was much more unlikely for Zarma participants to endorse this item compared with Hausa participants, relative to their overall perceived social acceptability of IPV. This item was dropped from all subsequent analyses. We recalibrated the unidimensional model with the remaining five items and found the original ordering of item difficulty was preserved even with the biased item removed (Table 3). No remaining items met the threshold for identification of DIF. EAP reliability for this 5-item measure was 0.74 and the Cronbach’s alpha was 0.82.

**Table 3:** Unidimensional 5-item partial credit model scale reliability and properties (Reaching Married Adolescents Study, 2016–2019, *n* = 559)

Items	Item response theory				CTT
	(EAP reliability: 0.740)				(Cronbach's *α*: 0.820)
	Item difficulty (SE)	Weighted mean square error fit (CI)	DIF: Zarma (vs. Hausa)	Point-biserial (response 0, 1, 2)	Item-total correlations
1. Use family planning	0.594 (0.074)	0.88 (0.86, 1.14)	− 0.044	− 0.65, 0.01, 0.68	0.85
2. Force sex	2.423 (0.093)	1.20 (0.84, 1.16)	− 0.230	− 0.43, 0.18, 0.40	0.62
3. Refuse sex	1.816 (0.082)	0.93 (0.86, 1.14)	− 0.026	− 0.64, 0.23, 0.59	0.80
4. Argue	1.459 (0.077)	1.11 (0.86, 1.14)	0.248	− 0.58, 0.11, 0.57	0.77
5. Go out	1.411 (0.077)	0.93 (0.87, 1.13)	− 0.008	− 0.67, 0.20, 0.62	0.82
	Variance (SE): 3.741 (0.224)				

*SE* standard error, *CI* confidence interval

### Multidimensional partial credit model

Based on our hypothesis that the scale contained two dimensions, we fit a two-dimensional PCM, as well as two consecutive unidimensional models for each dimension. In comparing the unidimensional model to the multidimensional model, we found that the latter resulted in the better fit (significant at *p* < 0.01) and that the EAP reliabilities for each dimension in the two-dimensional model were improved from those of the unidimensional models (Table 4), confirming our hypothesis and motivating the use of the two-dimensional model for subsequent analyses. Dimension 1 (Wifely sexual and reproductive duties) had an EAP reliability of 0.72 and variance of 3.75 (standard error (SE): 0.22) and Dimension 2 (Challenges husband authority) had an EAP reliability of 0.74 and variance of 4.85 (SE: 0.29). A moderate correlation between the two dimensions was found (*r* = 0.85), affirming the finding that the two dimensions are not measuring the same subconstructs but do complement one another in capturing the higher-order construct of IPV social norms. All items in the MRCML had adequate weighted mean squared error fits and no remaining DIF was detected (Table 5).

**Table 4:** Comparison of EAP reliabilities for each dimension across consecutive and multidimensional partial credit models (Reaching Married Adolescents Study, 2016–2019, *n* = 559)

Dimensions	Consecutive reliability	Multidimensional reliability	Consecutive Variance (SE)	Multidimensional variance (SE)
1. Wifely sexual and reproductive duties	0.679	0.717	3.714 (0.222)	3.714 (0.222)
2. Challenges husband authority	0.720	0.740	4.472 (0.267)	4.851 (0.290)
	Unidimensional reliability: 0.74

**Table 5:** Multidimensional 5-item partial credit model scale reliability and properties (Reaching Married Adolescents Study, 2016–2019, *n* = 559)

Items	Item response theory			
	Item difficulty (SE)	Weighted mean square error fit (CI)	DIF: Zarma (vs. Hausa)	Point-biserial (response 0, 1, 2)
1. Use family planning	0.558 (0.051)	1.16 (0.83, 1.17)	0.206	− 0.65, 0.01, 0.68
2. Force sex	2.429 (0.094)	1.08 (0.84, 1.16)	− 0.282	− 0.42, 0.19, 0.39
3. Refuse sex	1.810 (0.084)	0.87 (0.86, 1.14)	− 0.022	− 0.64, 0.25, 0.57
4. Argue	1.589 (0.082)	1.21 (0.85, 1.15)	− 0.032	− 0.57, 0.11, 0.57
5. Go out	1.535 (0.082)	0.87 (0.85, 1.15)	− 0.316	− 0.70, 0.22, 0.63
	Dimension correlation: 0.847			

*SE* standard error, *CI* confidence interval

In both the latent regression unidimensional and two-dimensional PCM for RC, levels of participant endorsement of IPV-ASRA social norms did not vary significantly based on whether the husband’s wife reported he had or had not ever perpetrated RC against her [unidimensional PCM: 0.44 logits (95% confidence interval [CI]: − 0.12, 1.00); 2-dimensional PCM, dimension 1: 0.25 logits (95% CI: − 0.35, 0.85) and dimension 2: 0.40 (95% CI: − 0.26, 1.06); results not in tables].

In contrast, in the latent regression for IPV, levels of participant endorsement of IPV-ASRA social norms varied significantly based on whether the husband’s wife reported he had or had not ever perpetrated IPV against her. We found that while IPV perpetration did not make a difference for Dimension 1 (Wifely sexual and reproductive duties) (0.120 logits, 95% CI: − 0.42, 0.66), the difference was statistically significant for Dimension 2 (Challenges husband authority); husbands who perpetrated IPV had 0.703 logits (95% CI: 0.11, 1.30) higher perceived social acceptability of IPV compared to those who did not perpetrate IPV, which is about one third of a standard deviation higher score. When we calibrated the model for the three subgroups (yes IPV, no IPV, missing data on IPV), the statistically significant differences between the intercept for Dimension 2 of those who had perpetrated IPV and those that did not was confirmed (Table 6). For Dimension 2, those who perpetrated IPV had an average logit score of 0.768 (95% CI: 0.44, 1.10) and those who had not perpetrated IPV had an average logit score of − 0.275 (95% CI: − 0.51, − 0.04). We also fit a latent regression unidimensional PCM for Dimension 2 alone and found weak ability to differentiate between those who had perpetrated IPV and those who had not (0.597 logits, 95% CI: − 0.02, 1.22), indicating the importance of including all five items in this scale and not reducing it down to only items in Dimension 2.

**Table 6:** Multiple group multidimensional partial credit model with delta dimensional alignment for intimate partner violence perpetration (Reaching Married Adolescents Study, *n* = 559)

	Yes IPV (*n* = 51)		No IPV (*n* = 443)		Missing IPV (*n* = 66)	
	*β*	95% CI	*β*	95% CI	*β*	95% CI
*Dimension 1 (wifely sexual and reproductive duties)*						
Intercept	0.324	(− 0.02, 0.67)	0.037	(− 0.15, 0.22)	0.301	(− 0.25, 0.85)
Model variance	1.571	(0.96, 2.18)	3.953	(3.43, 4.47)	5.229	(3.45, 7.01)
*Dimension 2 (challenges husband authority)*						
Intercept	0.768	(0.44, 1.10)	− 0.275	(− 0.51, − 0.04)	0.389	(− 0.17, 0.95)
Model variance	1.420	(0.87, 1.97)	6.320	(5.49, 7.15)	5.347	(3.52, 7.17)
Correlation of dimensions	0.885	(0.80, 0.93)	0.804	(0.77, 0.84)	0.892	(0.83, 0.93)

*CI* confidence interval, *IPV*  intimate partner violence

## Discussion

The IPV-ASRA Social Norms scale measures the latent construct of social norms regarding the perceived acceptability of IPV against wives to control her agency, sexuality, and reproductive autonomy. In all models, the scale demonstrated strong reliability, as well as internal structure and external validity. The items showed acceptable fit with the two-dimensional PCM, in which two subconstructs of IPV-ASRA social norms were represented: social acceptability of husband-perpetrated IPV if a wife is (1) not fulfilling her wifely sexual and reproductive duties, and (2) challenging her husband’s authority. Based on these findings, this brief 5-item IPV-ASRA Social Norms scale has strong potential for enhancing measurement of IPV social norms.

The IPV-ASRA Social Norms scale was associated with husbands’ perpetration of IPV against their wives, providing further evidence of validity and confirming the utility of this scale for understanding IPV behaviour. As social norms are understood to be a primary factor shaping patterns of IPV and RC behaviour within populations [24, 43], the IPV-ASRA Social Norms scale could be a critical tool for understanding contextual risk for IPV in a community and for evaluating the impact of programs intending to change IPV social norms. Specifically, Dimension 2 (Challenges husband authority) varied by IPV perpetration. This finding suggests that in this cultural and social context, the norms that sanction wives for behaviours that challenge her husband’s authority are more closely tied to the social norms that condone IPV, a finding that could be explored further as an opportunity for IPV prevention. It may be that wife behaviours that deviate from the norm of obedience to husbands are perceived as more threatening to the current gender norm structure and are therefore seen as more deserving of violent punishment from husbands to discipline this behaviour [14]. In contrast, social expectations of wives to bear children, by being sexually available to their husbands and fertile, may be perceived as less threatening to current social power structures and less linked to the norms that condone IPV. While fertility and procreation are highly valued in this cultural context and a very important part of social expectations of females [40], there may be less communal consensus on whether there are certain circumstances when it is acceptable for a wife to refuse sex or use family planning (e.g., if she already has had multiple or male children) or whether such a transgression warrants violence. Previous qualitative research in Sub-Saharan Africa has documented that wife beating is most acceptable for purposes of discipline, findings that are reflected by the stronger association between the challenging authority dimension of this scale and IPV [4, 33]. Future qualitative work could help shed additional light on the types of gender norm transgressions that are perceived to merit IPV-related punishment and the mechanisms shaping these norms within villages. Statisticians using this scale to understand how the latent construct of IPV-ASRA social norms relates to IPV behaviour will benefit from using a two-dimensional PCM.

This scale was not found to be associated with wife reports of husbands RC behavior. We suspect that this is likely due to the small number of husbands with wives reporting RC victimization and that large number of parameters in the PCM models, both of which reduce statistical power. This also could be a clear indication that the scale would benefit from more than one item specifically related to the social acceptability of reproductive autonomy that could be included in future iterations of the scale. Future research to develop an additional RC item that would complement this scale and be most appropriate in this cultural context is needed.

The reproductive autonomy item (item 1; family planning use without informing the husband) was an addition to the original ATT-IPV scale from which this IPV-ASRA Social Norms scale was developed. This item was the only item to be included in both dimensions of the scale, including the dimension that was associated with IPV behaviour (Dimension 1; Challenges husband authority). Our results demonstrate that the addition of this item is highly useful in understanding IPV-ASRA norms in this context and is likely an item worth including in subsequent use of this scale. Previous research in the region has identified covert use of family planning by women to be a strategy many young wives use to manage the conflict they may experience between their desire to delay pregnancy on the one hand and on the other, the strong social taboos against family planning use [47]. While commonly practiced, covert use of FP may be particularly risky in terms of potential husband perpetration of IPV, as evidenced by the way the perceived social norms accepting of violence in such a situation contributes to predicting IPV behaviour. Research from Niger and other settings has demonstrated a strong link between RC and IPV, stressing the importance of considering RC in understanding IPV [20, 38, 41]. Further social norm measurement development efforts would benefit from including RC-related norms and understanding how they interact, if at all, with IPV-related norms.

An additional key contribution of this scale is that it directly measures social acceptability of IPV behaviours and does so among men, those most likely to perpetrate these forms of violence. One previously identified IPV social norms scale, the Partner Violence Norms Scale, has shown an association with women who have experienced IPV victimization. That scale aims to measure the construct of traditional gender role expectations with only one item reflecting norms acceptable of IPV perpetration, a set of social norms more distally related to IPV. Additionally, the scale was assessed only among women and related to their IPV victimization, rather than among the men perpetrating violence. The IPV-ASRA Social Norms scale in the current study measures norms accepting of IPV perpetration to control wife agency, sexuality, and reproductive autonomy and was assessed among those whose behaviour is most relevant (i.e., those who perpetrated IPV), providing strong evidence of validity and utility.

While results support that the IPV-ASRA Social Norms scale is a strong measure with utility in IPV research, its primary limitation is that in this sample, it does not include enough items to capture the full continuum of the latent construct of IPV social norms; the scale contained primarily items that were severe or hard to endorse regarding perceived acceptance of IPV and lacks items that represent less severe perceptions of acceptance of IPV. The test information function graph suggests that the scale is best for populations of men with average and high perceived social acceptability of IPV (i.e., a location between about − 0.5 and 2 logits). Measure development research to expand this scale to cover more levels of the construct’s continuum would be useful to improve this scale in order to enable further differentiation of mens’ perceived social norms. Because the reproductive autonomy item was the “easiest” to endorse, the inclusion of more reproductive autonomy-related items might help expand coverage of the construct. Specifically, expanding the measure to include items representing RC social norms would be useful for understanding the norms supporting RC behavior among husbands, and their interaction with IPV social norms. This should be done by triangulating qualitative and quantitative data from this population to inform which additional items are most relevant. In the scale’s current form, the middle response option of “somewhat agree” may have been more “difficult” to endorse than a more neutral wording of this response option (e.g., “neither agree nor disagree”), which may have contributed to the skewed coverage of the construct. Further cognitive interviews with this population around interpretation of this three-option Likert scale is needed. The brevity of the current version of this scale, however, is a strength in studies where participant burden is already high or in epidemiological studies, where measures typically need to be limited in length, so the addition of a limited number of well-constructed items is recommended. Lastly, there was substantial loss-to-follow-up in this wave of data collection for husbands that may have led to selection bias in this sample. Moving forward, the generalizability of the findings for this scale will be strengthened as it is tested in more diverse, representative samples of men from this cultural context.

## Conclusion

This IPV-ASRA Social Norms scale is a short, practical measure with strong reliability and validity evidence and is associated with men who perpetrate IPV. To date, very few measures of IPV social norms are available, and none, to our knowledge, have shown statistically significant associations with male IPV perpetration. This scale is concise and builds on a widely accepted and utilized measure of individual attitudes about justification for IPV (ATT-IPV) included in the DHS, and, with additional testing in other cultural contexts, could be a natural and useful addition to DHS-related efforts to understand the context of IPV. Enhancing current approximations of IPV social norms that simply aggregate individual attitudes, this scale directly measures social norms of IPV behaviour and could help elucidate pathways through which social norms may be impacting IPV behaviour. Moreover, as social norms are increasingly becoming the focus of IPV prevention efforts, the IPV-ASRA Social Norms scale could be used to examine areas of high need for social norms-focused prevention and to measure the impact of such efforts.

## Supplementary Information

**Additional file 1.** Reproductive coercion survey items asked of adolescent wives living in Dosso, Niger. Table of survey questions used to measure reproductive coercion victimization.

## Data Availability

The data that support the findings of this study are available from the corresponding author, SB, upon reasonable request.

## Abbreviations

ATT-IPV: Attitudes about intimate partner violence scale
DHS: Demographic and Health Survey
DIF: Differential item functioning
EAP: Expected a-posteriori
IPV: Intimate partner violence
IPV-ASRA: Intimate partner violence to control wife agency, sexuality, and reproductive autonomy
MRCML: Multidimensional random coefficients multinomial logit
PCM: Partial Credit Model
RC: Reproductive coercion
RMA: Reaching Married Adolescents Study in Niger
