# Supplementary material for: Measuring social norms of intimate partner violence to exert control over wife agency, sexuality, and reproductive autonomy: an item response modelling of the IPV-ASRA scale
Source: Reprod Health. 2023 Jun 14;20:90. doi: 10.1186/s12978-023-01632-w (PMC10265906; doi:10.1186/s12978-023-01632-w)
Supplement: Supplementary file 1 — Additional file 1. Reproductive coercion survey items asked of adolescent wives living in Dosso, Niger. Table of survey questions used to measure reproductive coercion victimization. [file 12978_2023_1632_MOESM1_ESM.docx]

| **Additional file 1.** **Reproductive coercion survey items asked of adolescent wives living in Dosso, Niger** | | |
| --- | --- | --- |
|  | **Survey Items** | **Response options (Bold** indicates a response counted as an affirmative response to husband RC**)** |
| 1 | Have any of the following people ever pressured you, made you feel badly, or treated you badly for wanting to use a family planning method to delay or prevent pregnancy? | **1 Yes, husband** 2 Yes, in-laws (husband's family) **3 Yes, husband and in-laws** 4 No, no one 997 Other  998 Don’t know |
| 2 | Has your husband ever tried to force or pressure you to become pregnant? | **1 Yes** 0 No 998 Don't know 999 Decline to answer |
| 3 | Has your husband ever hidden, destroyed or taken away your family planning method (e.g., Pills)? |  |
| 4 | Has your husband ever demanded that your family planning method be removed for example, implant or IUD? |  |
| 5 | Has your husband ever stopped you from going to a casa sante (health center) to obtain family planning? |  |
| 6 | Has your husband ever said he would leave you if you did not get pregnant? |  |
| 7 | Has your husband ever threatened to hurt or beat you if you tried to prevent or delay getting pregnant? |  |
| 8 | Has your husband ever told you that you could not use family planning because you did not have enough sons? |  |
| 9 | Has your husband ever insulted you, yelled at you, or made you feel badly for using or wanting to use family planning? |  |
